# Supplementary material for: Fuzzy supertertiary interactions within PSD-95 enable ligand binding
Source: eLife. 2022 Sep 7;11:e77242. doi: 10.7554/eLife.77242 (PMC9581536; doi:10.7554/eLife.77242)
Supplement: Source code 1. — Included are scripts to align 2 channel FRET images, extract time traces for intensity maxima, manually select single molecule traces and calculate FRET efficiency from selected molecules. A README file is added to aid in implementation of the scripts with MATLAB. [file elife-77242-code1.zip › BowenLab_DataAnalysis/220818 README.docx]

FRET Analysis Method from:

“**Fuzzy Supertertiary Interactions within PSD-95 Enable Ligand Binding**”

George L. Hamilton*, Nabanita Saikia*, Sujit Basak, Franceine S. Welcome, Fang Wu, Jakub Kubiak, Changcheng Zhang, Yan Hao, Claus A.M. Seidel, Feng Ding^§^, Hugo Sanabria^§^, Mark E. Bowen^§^

This protocol extracts FRET efficiency from time traces of single molecules taken from data acquired with a 512x512 pixel emCCD camera. This protocol is for use with an image splitter to separate donor and acceptor intensity with donor on the left half and acceptor on the right half of the image. The associated MATLAB script package requires that movies are in the Multi-Image TIFF file format.

A complete data requires a calibration image for the donor-acceptor channel alignment, which is a 10-frame movie of fluorescent beads on the order of the diffraction limit (e.g. Thermo Scientific R300 was used in this work) excited by the donor laser with emission in both donor and acceptor channels. Our data acquisition sequence alternates the laser wavelength to excite Acceptor-Donor-Acceptor in sequence (e.g. Figure 4, Figure Supplement 1). The First Acceptor Excitation Phase is to identify acceptor-labeled molecules. The Donor Excitation Phase is to capture the FRET efficiency data. The Final Acceptor Excitation Phase is to confirm acceptor bleaching. In this paper the number of frames of each excitation period was 10 red-500 green-50 red frames.

These scripts are an implementation of our published analysis methods ^1^. **BowenLabMovieProcessor** allows the user to select from 3 different background subtraction methods: the local *median* using 16 x 16-pixel squares, the SPARTAN method adapted from the Blanchard lab ^2^, or the local *minimum* using 16 x 16-pixel squares. For this paper, we used the local median. The script finds the highest intensity pixels within the image and outputs their intensity over time. These scripts also allow the user to select from 2 different intensity calculation methods: sum of the top four brightest pixels from a single molecule or sum a 3 x 3-pixel area centered on the maxima from a single molecule. This script allows manual trace viewing and molecule selection from the *entire* dataset of intensity maxima and returns a 10 frame average FRET efficiency for each selected molecule. The data can be displayed in 2 ways. For FRET studies, both donor and acceptor appear in the same plot above a plot of the calculated FRET efficiency over time. Alternately, the intensity for each channel can be plotted in Separate Intensity time traces without displaying FRET efficiency. Using the **BowenLabMovieProcessor** for trace analysis is recommended for a first look at the data but is time consuming as many intensity maxima may not be single molecules.

Criteria for Identifying Single Molecules from Time Traces

1. Trace shows stable Acceptor Intensity, which is consistent with that of a single dye under the experimental conditions, during the entirety of the First Acceptor Excitation Phase.
2. Both the Acceptor and Donor dyes must show a Single-Step Photobleaching event to baseline during Donor Excitation Phase.
3. For γ-correction of FRET efficiency, which was used in this paper, traces must show an Anti-Correlation between acceptor and donor intensities during at the time of acceptor photobleaching.
4. For best results, selected
5. traces should have a relatively high intensity to insure good signal to noise levels.

To speed manual trace selection, **BowenLab_PruningTraceViewer** automatically filters out traces that fail to meet the first two criteria. Finally, selected single molecules are output as individual files, which are analyzed with the **BowenLab_FRET_Analysis** script package to extract the γ-corrected FRET efficiencies. These scripts use a Gaussian derivative kernel algorithm adapted from the Weninger lab ^3^.

**smTIRF Data Analysis Workflow:**

1. ***Find the Offsets between Donor and Acceptor Channels with a Calibration Image.***
   1. The Calibration Image is a 10 frame movie of fluorescent beads.
   2. Run **BowenLab_Find_Bead_Offset.m**
   3. Follow prompts to load the Calibration Image
   4. The offsets (X, Y) in pixels is displayed in the command window
   5. The offsets are also output to a text file*“OFFSET.txt”* in the current directory.
2. ***Extract the Intensity Time Traces from multi-image TIFF movies.***
   1. Manually enter the (X, Y) offsets acquired in step 1 into the **BowenLabMovieProcessor.m** mfile on lines 23 and 24.
   2. Run the script and follow prompts to load the data file in the form of a multi-image TIFF movie.
   3. Follow the prompts to enter the laser excitation sequence information.
   4. Follow the prompts to choose the background subtraction method.
   5. Follow the prompts to choose the intensity calculation method
   6. The script will output intensity time traces for all intensity maxima in a single *“*.traces”* file that shares the name of the original movie file.
   7. Follow the prompts to choose “yes” to continue to view traces for all intensity maxima, or “no” to exit and view only filtered traces.
   8. If continuing, follow the prompts to select FRET as the Trace View mode.
3. ***Select traces***
   1. Run **BowenLab_PruningTraceView.m**
   2. Follow the prompts to enter the laser excitation sequence information.
   3. Follow the prompts then load the **.traces* file from Step 2.6.
   4. Manually examine traces selecting those meeting the experimental criteria.
      1. Press Enter to pass traces that are not to be included in the analysis.
      2. Press “s” to save the selected traces
      3. Press “b” to back up one trace.
      4. Press “q” to quit early. The script will terminate after all traces have been examined.
   5. Each selected intensity trace is saved as individually files in the Igor (Wavemetrics) file format *(*.itx)* with the movie name and the molecule number.
   6. This script will output some cursory analysis for selected traces into multiple text files, which based on the first 10 frames of the movie.
      1. *avg_FRET.txt* contains the time-averaged FRET efficiency.
      2. *avg_D.txt* contains the time-averaged intensity for the donor channel.
      3. *avg_A.txt* contains the time-averaged intensity for the acceptor channel.
      4. *s2n_D.txt* contains the time-averaged signal to noise for the donor channel (mean/stdev).
      5. *s2n_A.txt* contains the time-averaged signal to noise for the acceptor channel (mean/stdev).
4. ***Calculate FRET efficiency for selected traces***
   1. Run **BowenLab_FRET_Analysis.m.**
   2. Follow the prompts to enter the laser excitation sequence information.
   3. Manually examine traces selecting those with correctly adjusted intensity baseline and correct identification of the photobleaching times.
      1. Press “s” to save the selected trace and use the data to calculate FRET efficiency.
      2. Press “m” to manually select the frame for acceptor photobleaching using the mouse should the automated edge detection fail.
      3. Press Enter to pass traces that are not to be included in the analysis should the automated fitting prove uncorrectable.
      4. Press “b” to back up one trace.
      5. Press “p” to save representative traces for publication.
      6. Press “q” to quit early. The script will terminate after all traces have been examined.
   4. The results of the analysis will be output into multiple text files.
      1. *pwFRET.txt*: contains the “point-wise”, gamma-corrected FRET efficiency, which is calculated for each movie frame for all selected traces.
      2. *Acceptor.txt*: contains the time-averaged acceptor intensity for each selected trace, which is the average intensity prior to the acceptor photobleaching.
      3. *Donor*.txt: contains the time-averaged donor intensity for each selected trace, which is the average intensity prior to acceptor photobleaching.
      4. *rawFRET.txt*: contains the time-averaged FRET efficiency for selected trace prior to acceptor photobleaching.
      5. *working.mat*: contains all variables in the MATLAB workspace during the analysis for diagnostic purposes.

**References**

1. McCann, J.J., Choi, U.B., Zheng, L., Weninger, K. & Bowen, M.E. Optimizing methods to recover absolute FRET efficiency from immobilized single molecules. *Biophys J* **99**, 961-70 (2010).

2. Juette, M.F. et al. Single-molecule imaging of non-equilibrium molecular ensembles on the millisecond timescale. *Nature Methods* **13**, 341-344 (2016).

3. Sass, L.E., Lanyi, C., Weninger, K. & Erie, D.A. Single-molecule FRET TACKLE reveals highly dynamic mismatched DNA-MutS complexes. *Biochemistry* **49**, 3174-90 (2010).
