# Supplementary material for: Fuzzy supertertiary interactions within PSD-95 enable ligand binding
Source: eLife. 2022 Sep 7;11:e77242. doi: 10.7554/eLife.77242 (PMC9581536; doi:10.7554/eLife.77242)
Supplement: Transparent reporting form [file elife-77242-transrepform1.docx]

***eLife’s* transparent reporting form**

We encourage authors to provide detailed information *within their submission* to facilitate the interpretation and replication of experiments. Authors can upload supporting documentation to indicate the use of appropriate reporting guidelines for health-related research (see [EQUATOR Network](http://www.equator-network.org/%20)), life science research (see the [BioSharing Information Resource](https://biosharing.org/)), or the [ARRIVE guidelines](http://www.plosbiology.org/article/info:doi/10.1371/journal.pbio.1000412) for reporting work involving animal research. Where applicable, authors should refer to any relevant reporting standards documents in this form.

If you have any questions, please consult our Journal Policies and/or contact us: [editorial@elifesciences.org](mailto:editorial@elifesciences.org).

**Sample-size estimation**

- You should state whether an appropriate sample size was computed when the study was being designed
- You should state the statistical method of sample size computation and any required assumptions
- If no explicit power analysis was used, you should describe how you decided what sample (replicate) size (number) to use

Please outline where this information can be found within the submission (e.g., sections or figure legends), or explain why this information doesn’t apply to your submission:

The single molecule experiments report all collected instances of single dye pairs containing one acceptor and one donor that meet the criteria outlined in materials and methods. In TIRF experiments, molecules are accumulated until the position of the predominant peak in the accumulated histogram (with constant 0.05 FRET unit binning) becomes insensitive to molecule number, which is typically 50-100 molecules depending on the complexity of the distribution. In confocal single-molecule confocal experiments, we observed an average over 3600 FRET-exhibiting molecules per FRET experiment. Additional details about data analysis can be found in the materials and methods.

**Replicates**

- You should report how often each experiment was performed
- You should include a definition of biological versus technical replication
- The data obtained should be provided and sufficient information should be provided to indicate the number of independent biological and/or technical replicates
- If you encountered any outliers, you should describe how these were handled
- Criteria for exclusion/inclusion of data should be clearly stated
- High-throughput sequence data should be uploaded before submission, with a private link for reviewers provided (these are available from both GEO and ArrayExpress)

Please outline where this information can be found within the submission (e.g., sections or figure legends), or explain why this information doesn’t apply to your submission:

We measured 12 independent FRET variants of full-length PSD-95, and 6 additional FRET variants for the PSG fragment. For each, a single sample is used since the purification and labelling reactions are not considered a significant variable in this study. Each sample was expressed once then labelled and measured in 2 (or 3) different laboratories using different fluorescent dyes and different data collection modalities as described in materials and methods. The unprecedented comparison between smFRET methodologies is a highlight of the manuscript (Figure 4). For smTIRF, we make 3 replicate surface attachment preparations, which can affect data quality, and from each record data from 5 or more fields of view depending on surface density. In confocal experiments, each observed event is an independent measurement of a FRET-exhibiting molecule during a single collection period. Additional details can be found in the materials and methods.

**Statistical reporting**

- Statistical analysis methods should be described and justified
- Raw data should be presented in figures whenever informative to do so (typically when N per group is less than 10)
- For each experiment, you should identify the statistical tests used, exact values of N, definitions of center, methods of multiple test correction, and dispersion and precision measures (e.g., mean, median, SD, SEM, confidence intervals; and, for the major substantive results, a measure of effect size (e.g., Pearson's r, Cohen's d)
- Report exact p-values wherever possible alongside the summary statistics and 95% confidence intervals. These should be reported for all key questions and not only when the p-value is less than 0.05.

Please outline where this information can be found within the submission (e.g., sections or figure legends), or explain why this information doesn’t apply to your submission:

Determination of confidence intervals for FRET-derived parameters from single-molecule data was carried out by using the F-test for the ratio of Chi-squared statistics to independently obtain the 95% confidence interval for each fit parameter. To obtain confidence intervals for structure classification, we used the standard deviation of the distances from repeated refitting of subsampled data (a.k.a. bootstrapping) from fluorescence decay histograms, which were each refit an average of 19 times.

(For large datasets, or papers with a very large number of statistical tests, you may upload a single table file with tests, Ns, etc., with reference to sections in the manuscript.)

**Group allocation**

- Indicate how samples were allocated into experimental groups (in the case of clinical studies, please specify allocation to treatment method); if randomization was used, please also state if restricted randomization was applied
- Indicate if masking was used during group allocation, data collection and/or data analysis

Please outline where this information can be found within the submission (e.g., sections or figure legends), or explain why this information doesn’t apply to your submission:

Samples were allocated in two groups: full length PSD-95 and the truncated PSG fragment.

**Additional data files (“source data”)**

- We encourage you to upload relevant additional data files, such as numerical data that are represented as a graph in a figure, or as a summary table
- Where provided, these should be in the most useful format, and they can be uploaded as “Source data” files linked to a main figure or table
- Include model definition files including the full list of parameters used
- Include code used for data analysis (e.g., R, MatLab)
- Avoid stating that data files are “available upon request”

Please indicate the figures or tables for which source data files have been provided:

Data and analysis of confocal single-molecule experiments in Figures 3, 4 and 5 are publically available (10.5281/zenodo.6001898). This repository also includes data from Figure 6 for accessible volume simulations performed on DMD simulations to obtain interdye distances. Source data used to construct smTIRF histograms are available as Source Data for Figure 4. DMD simulation data shown in Figures 6 and 10 are publically available (https://dlab.clemson.edu/research/PSD95-PSG/)
